# Knowledge, attitude and practice of tobacco smoking by medical students in Riyadh, Saudi Arabia

**DOI:** 10.4103/1817-1737.65044

**Published:** 2010

**Authors:** Ali I. Al-Haqwi, Hani Tamim, Ali Asery

**Affiliations:** Family and Community Medicine, King Saud Bin Abdul Aziz University for Health Sciences, Riyadh, Saudi Arabia; 1 Epidemiology and Biostatistics, King Saud Bin Abdul Aziz University for Health Sciences, Riyadh, Saudi Arabia; 2 Department of Pediatrics Gastroenterology, College of Medicine, King Fahad Medical City, Riyadh, Saudi Arabia

**Keywords:** Medical students, Saudi Arabia, smoking

## Abstract

**BACKGROUND:**

Tobacco consumption is associated with considerable negative impact on health. Health professionals, including future doctors, should have a leading role in combating smoking in the community.

**OBJECTIVES:**

The aims of the study were to assess the prevalence of smoking among medical students of newly established medical colleges in Riyadh city, the capital of Saudi Arabia, as well as to assess students' attitude, practice and their knowledge on the risk factors of tobacco consumption.

**METHODS:**

A cross-sectional, questionnaire-based study of students from two medical colleges in Riyadh, Saudi Arabia was carried out. The questionnaire used was anonymous, self-administered and developed mainly from Global Adult Tobacco Survey (GATS).

**RESULTS:**

A total of 215 students participated in this study. Forty students (19%) indicated that they smoke tobacco at the time of the study. All of them were males, which raise the prevalence among male students to 24%. Tobacco smoking was practiced by males more than females (P value <0.0001) and by senior more than junior students (<0.0001). About 94% of the study sample indicated that smoking could cause serious illnesses. About 90% of the students indicated that they would advice their patients to quit smoking in the future and 88% thought that smoking should be banned in public areas. Forty-four students (20%) thought that smoking has some beneficial effects, mainly as a coping strategy for stress alleviation.

**CONCLUSION:**

Despite good knowledge about the hazards of tobacco consumption, about 25% of the medical students in this study continue to smoke. The main reported reasons should be addressed urgently by policy-makers. Special efforts should be taken to educate medical students on the effective strategies in managing stress during their study as they thought that tobacco smoking could be used as a coping strategy to face such a stress.

The general negative impact of tobacco smoking on health is significant. Currently, the mortality due to tobacco smoking has been estimated to be more than 5 million deaths annually,[1] which is expected to double by 2020.[2] This significant impact is not only due to morbidity and mortality, but also attributed to the social and the economic cost of smoking.[3] Reports have indicated that the real impact of tobacco smoking could be underestimated because of high level of exposure of “secondhand” smoke, increased smoking among youths and the increase in smoking among nonsmokers.[4]

An international review showed that the prevalence of tobacco smoking varies greatly among medical students from one country to another. Its prevalence varies among male medical students from 3% in the United States to 53% in Japan.[5] To a lesser extent, variation has been reported regionally. Smoking ranges from 15% to 35% in three different regional studies among university students.[6–8] In Saudi Arabia, the overall prevalence of smoking in both the general population and university students, ranges from 21% to 25%.[9–11] Tobacco smoking was reported to be practiced by approximately 13% of male medical students.[12,13] To the best of the authors' knowledge, there are no published data about smoking among students of the newly established medical colleges that adopt Problem Based Learning curriculum.

The health professionals, including future doctors, play an important role in educating patients about the hazards of tobacco smoking, providing advice, support and motivation to patients to quit smoking. Thus, their views and attitude are of great importance to be determined before implementing any anti-tobacco measures.

This study was carried out to determine the prevalence of smoking among medical students from newly established colleges in Riyadh city, the capital of Saudi Arabia. Students' attitude, practice and their knowledge of the risk factors of tobacco consumption were also sought.

## Methods

This is a cross-sectional study which was carried out during the month of June 2009 and involved students from two medical colleges in Riyadh, the capital of Saudi Arabia.

These two colleges were selected in order to have a homogenous sample of students as they were the only colleges adopting “Problem Based Learning” curriculum at the time of the study. All the students of these two colleges were invited to participate in the study by filling an anonymous self-administered questionnaire.

A questionnaire was developed mainly from Global Adult Tobacco Survey (GATS) and was used in our study. This instrument aims to assess main issues related to tobacco consumption, which has been developed and validated by collaboration of Centers of Disease Control and Prevention (CDC), World Health Organization, CDC Foundations, John Hopkins Bloomberg School of Public Health (JHSPH) and Research Triangle Institute (RTI).[14]

The questionnaire was written in English and includes information about demographic data of participants including their study level and GPA. Study level stands for the academic stage of the students which ranges from level 1, junior to level 5, and senior level. GPA stands for Grade Point Average, which reflects the academic performance of students.

The questionnaire included as well information about students' smoking practices, their knowledge and attitude toward smoking. In order to achieve the highest possible accuracy, participation in this study was voluntary and the questionnaire was anonymous and self-administered.

For the purpose of this study, smoking status was defined as regular or occasional cigarette or water pipe smoking at the time of the study. Nonsmokers are those who never smoke or were ex-smokers.

Data was coded, entered and analyzed using the Statistical Package for Social Sciences (SPSS), version.[17] Descriptive analyses were done to summarize information by calculating the number and percent for categorical variables, whereas the mean and standard deviation (s.d.) were calculated for continuous variables. Chi-squared test was used to measure difference in the prevalence of smoking between different groups of students. A P value less than 0.05 was considered statistically significant.

The proposal of this study had been revised and approved by the ethics committee of King Abdullah International Research Center.

## Results

A total number of 215 out of 330 students participated in this study, which makes a response rate of 65%. The mean age of the participants was 21 years (s.d. = 3). Male students represented 77% and more than 90% were single. Other characteristics of the study sample are shown in Table 1.

**Table 1:** Characteristics of participants

	Number (n)	Percentage (%)
Sex		
Male	165	77
Female	50	23
Marital status		
Single	200	93
Married	15	7
Study level		
1	86	40
2	35	17
3	27	13
4	46	22
5	17	8
GPA		
Excellent	49	23
Very good	78	36
Good	54	25
Fair	16	7

Forty students (19%) indicated that they used to smoke tobacco at the time of the study. All of them were males, which raised the prevalence among male students to 24%. The differences between characteristics of smokers and nonsmokers are shown in Table 2.

**Table 2:** Association between students' characteristics and smoking status

	Smokers n (%)	Nonsmokers n (%)	*P* value
Sex			
Male	40 (24)	125 (76)	<0.0001
Female	0 (0)	50 (100)	
Marital status			
Single	36 (18)	163 (82)	0.41
Married	4 (27)	11 (73)	
Study level			
1	8 (9)	78 (91)	<0.0001
2	2 (6)	33 (94)	
3	8 (7)	19 (93)	
4	13 (28)	33 (72)	
5	9 (53)	8 (17)	
GPA			
Excellent	6 (12)	43 (82)	0.477
Very good	16 (21)	62 (79)	
Good	14 (26)	40 (74)	
Fair	2 (13)	14 (16)	

The practice of smoking was higher among males with a significant statistical difference. Tobacco smoking was practiced by males more than females (*P* value <0.0001) and by senior students more than junior students (<0.0001).

About 94% of the study sample indicated that smoking could cause serious illnesses. The students also indicated, as shown in Table 3, that smoking is related to major chronic diseases, especially lung cancer and heart diseases, but to a lesser extent, to sexual dysfunction, as approximately a third of the students did not know if smoking could cause any sexual dysfunctions.

About 90% of the students indicated that they would advise their patients to quit smoking in the future, and 88% thought that smoking should be banned in public areas.

**Table 3:** Students' responses to the relationships between smoking and certain diseases

Diseases	Yes n (%)	No n (%)	Don' know n (%)
Stroke	180 (83)	5 (2)	27 (13)
Heart diseases	192 (89)	4 (2)	17 (8)
Lung cancer	202 (94)	3(1.4)	8 (4)
Gum diseases	182 (84)	5 (2)	25 (12)
Hypertension	173 (80)	10 (5)	26 (12)
Sexual dysfunction	126 (58)	21 (10)	62 (29)

The influence of friends and peer pressure was perceived as the main reason for smoking as shown in Table 4, followed by stress and the effect of promoting smoking through media.

**Table 4:** Students' perception on the main causes of smoking

	Responses	
	Number (n)	Percentage (%)
Friends	164	80
Stress	56	26
Media	29	13

Forty-four students (20%) thought that smoking has some beneficial effects. The main effect was related the use of smoking as a coping strategy for stress alleviation. Moreover, students thought that smoking plays a role in preventing some diseases like Parkinsonism and viral diseases. Other perceived benefits of smoking are shown in Table 5.

**Table 5:** Benefits of smoking

	Responses	
	Number (n)	Percentage (%)
Relief stress	24	69
Protect against some diseases	5	14
Increase concentration	3	8
Social benefits	3	8

## Discussion

Health professionals, including future doctors, have a leading role in combating smoking in the community. Thus, it is of great importance to determine their views and attitude toward this problem.

This study has demonstrated that the prevalence of smoking among medical students is 19% and rises to reach up to 24% among males. The prevalence of smoking among students of newly established problem based medical colleges was comparable to that of previously reported data among students of colleges adopting traditional medical curriculums.[7,13,14]

It has been reported that compared to conventional teaching methods, Problem Based Learning particularly promotes students' active learning, interpersonal skills and problem solving abilities.[15] In addition to this, Problem Based learning increases students' motivation and enjoyment.[16] Such advantages of Problem Based Learning are expected to make students less stressed and less involved in stress-related behaviors like tobacco smoking.

The findings of this study confirm similar smoking prevalence among students of newly established medical colleges, and their collogues from other medical colleges.

Tobacco smoking was found to be significantly higher among senior students compared to those in first 2 years. Similar findings have been reported,[8–10,17] as the risk of tobacco consumption increases with students' progression. This is probably due to increased stress faced by the students with their progression, over the years. There was no effect of marital status and academic achievements on tobacco use in this study; however, other studies showed that single and poor performing students tend to smoke more.[8]

Students showed high awareness of the hazards of smoking especially in relation to the significant role of smoking in lung cancer and cardiovascular diseases. Students appreciated the risks of passive smoking and agree with the decisions of banning it in public areas. They also have indicated their willingness in taking a positive and active role to reduce the tobacco consumption of their future patients.

The effect of the influence of peers as a major determinant of tobacco smoking was comparable to that reported in regional and local studies.[6,14] Students indicated that tobacco is consumed to overcome the stress experienced during their studies. The influence of peers and the role of life-stressors in general are particularly important as they were perceived by medical students to contribute to more serious behaviors such as alcohol and substance use and abuse.[18] This finding should be taken very seriously as students use tobacco smoking as a stress coping strategy.

In conclusion, despite good knowledge on the hazards of tobacco consumption, about 25% of the medical students in this study continue to smoke. The main reasons reported are due to peers pressure and stress. This is supported by the finding of this study that the prevalence of tobacco consumption is significantly higher among senior students.

Students showed positive attitude toward minimizing passive smoking through their support of banning smoking in public areas as well as their willingness to discuss and advise their patients to quit smoking.

Given their vital role as future physicians and role models, more effective approaches to help reduce tobacco consumption among medical students are needed.

Efforts should be taken to educate medical students on effective strategies in managing stress during their course, as they thought that tobacco smoking could be used as a coping strategy to face such stress. In this regard, multiple and regular “stress coping strategies” sessions could be organized for medical students to help them to cope with life stressors and to minimize the possibility of smoking and probably more serious behaviors such as use of alcohol or other substances.

It may be appropriate to consider implementing counseling programs to support students, especially during difficult periods of their medical course.
